# Internal and External Loads of Young Elite Soccer Players during Defensive Small-Sided Games

**DOI:** 10.5114/jhk/162027

**Published:** 2023-04-20

**Authors:** Alberto Rabano-Muñoz, Luis Suarez-Arrones, Bernardo Requena, Jose A. Asian-Clemente

**Affiliations:** 1 Performance Department, Real Betis Balompié, Seville, Spain.; 2 Department of Sport Sciences, Universidad Pablo de Olavide, Seville, Spain.; 3 Performance Department, Football Science Institute, Granada, Spain.

**Keywords:** small-sided games, global positioning system (GPS), external load, heart rate, internal load

## Abstract

The aim of this study was to evaluate the effects of different time periods on the internal and external loads of soccer players during small-sided games (SSGs). Seventeen young soccer players performed a SSG of 5 vs. 5 + 5 with 2 floaters, where two teams had possession of the ball, and one had to recover it. With established periods of 30 s (SSG_30_), 1 min (SSG_1_) or 2 min (SSG_2_), teams participated in a defensive role for these periods of time. Total distance covered, moderate speed running distance, high speed running distance, sprint running distance, accelerations, decelerations and Player Load were monitored using global positioning systems devices. The maximal heart rate and modified training impulse were monitored using heart rate monitors. The rating of perceived exertion (RPE) was also measured. Data showed a small increase between SSG_30_ and SSG_1_ in Player Load (ES = −0.35; p < 0.01), and a small increase in high speed running (ES = −0.41; p < 0.05) and sprinting (ES = −0.47; p < 0.01) between SSG_30_ and SSG_2_. Also, SSG_1_ showed a small increase in sprinting (ES = −0.57; p < 0.01) and accelerations (ES = −0.37; p < 0.05) with respect to SSG_2_. In addition, SSG_2_ showed a small increase in the RPE with respect to SSG_30_ (ES = 0.46; p < 0.05). The results indicate that shorter defensive periods in SSGs increased high speed running, while longer defensive periods promoted greater perceived exertion. Manipulation of the duration of defensive periods in SSGs is a variable that should be considered in soccer training.

## Introduction

Small-sided games (SSGs) are among the tasks most often used by soccer coaches during training (Halouani et al., 2014; Sarmento et al., 2018). SSGs represent modified games played on reduced pitch areas, with a smaller number of players than competition matches, and adapted rules (Owen et al., 2020). The popularity of these soccer drills has grown in recent years due to their suitability for developing technical, tactical and physical aspects in a soccer-specific context at the same time (Jones and Drust, 2007; Owen et al., 2014), increasing motivation, and improving efficiency during training sessions (Casamichana et al., 2012). Furthermore, SSGs induce an integrated approach for soccer training because they present advantages in terms of specificity of movements and inclusion of decision making (Hammami et al., 2018), and they allow to increase variability between sessions, ensuring that game movement patterns are replicated, as well as the physiological and technical demands of competition (Clemente et al., 2021).

In order to reproduce competition demands, multiple formats of SSGs have been developed and reported in the scientific literature (Aguiar et al., 2012; Hill-Haas et al., 2011; Sarmento et al., 2018). Some of the most popular variables investigated are pitch size, the number of players and the multiple combinations of them (Casamichana and Castellano, 2010; Kelly and Drust, 2009), inclusion of goalkeepers (Mallo and Navarro, 2008), inclusion of floaters, numerical imbalances (Lacome et al., 2018; Rabano-Munoz et al., 2019), and encouragement from coaches (Rampinini et al., 2007). Another key variable during the design of an SSG is the duration format, which includes the lengths of the SSG, the rest intervals between sets, and the number of sets. SSG duration is extensively manipulated by coaches in their daily training, and has been shown to be a determinant factor for players’ loads (Halouani et al., 2014; Sarmento et al., 2018). Several previous studies have shown that long and continuous SSG duration formats generated greater player internal loads (Clemente et al., 2019a; Hill-Haas et al., 2009; Tessitore et al., 2006) in contrast to intermittent and short SSG formats, which showed greater total distance covered and more high speed running (Casamichana et al., 2013; Clemente et al., 2019a; Fanchini et al., 2011). Clemente et al. (2019a) analysed the variations in internal and external loads in a 5 vs. 5 SSG format with two different duration routines (3 x 6 min and 6 x 3 min), suggesting that longer sets increase the perception of effort, and contribute to a large decrease in running distances. Casamichana et al. (2013) used the same format of 5 vs. 5 SSG in a continuous format of 16 min, a 4 x 4 min format and a 2 x 8 min format, concluding that short duration formats resulted in a greater total distance covered and higher stability in physiological responses.

However, more research is still needed with regard to duration formats in SSGs. Previous studies have been carried out with players participating in defensive and offensive phases of the game for variable and uncontrolled periods. Considering that the behaviour of players is different in the defensive and offensive phases (Silva et al., 2014), and tactical decisions modulate physical actions employed by players (Schuth et al., 2016), defensive roles during the game could be more demanding that offensive roles (Castellano et al., 2022). Thereby, it is very frequent in soccer to design defensive SSGs where players must be in a defending mode for a specified period of time. However, although they are extensively used, to the best of our knowledge the consequences of these defensive drills and the responses of soccer players to them are still unknown. For this reason, the aim of this study was to evaluate the effects of specific time periods of defensive phase play on the internal and external loads of soccer players during the same SSG. The hypothesis was that the duration of the defensive period would affect the external and internal loads of players. Furthermore, shorter defensive periods would be more demanding in external loads and longer defensive periods would increase internal loads.

## Methods

### Participants

Seventeen young soccer players (age: 15.2 ± 0.3 years; body height: 171.4 ± 6.5 cm; body mass: 62.5 ± 7.5 kg; YYIRT1 performance: 2365 ± 467 m) from an elite academy of a Spanish first division club participated in this study. Players participated in four training sessions and one competitive match (Sunday) per week, and were highly familiarized with SSGs as these were very frequently used in their daily training. All players who participated in the study were outfield players. These data were created as a condition of monitoring in which players’ activities were measured during the competitive season (Winter and Maughan, 2009), so that the ethics committee clearance was not required. The study conformed nevertheless to the recommendations of the Declaration of Helsinki. Participants were informed of the design and gave their consent before the start of the study.

### Measures

External loads were monitored using global positioning system devices operating at a sampling frequency of 10 Hz (Catapult Vector S7, Catapult Sports, Australia). The reliability and accuracy of these devices have been validated previously (Johnston et al., 2014). Variables measured were: total distance covered, distance covered between 14.0 and 17.9 km•h^-1^ (moderate speed running), distance covered between 18.0 and 20.9 km•h^-1^ (high speed running), and distance covered above 21.0 km•h^-1^ (sprinting). These thresholds have been widely used previously (Asian-Clemente et al., 2022; Casamichana et al., 2012; Suarez-Arrones et al., 2015). Simultaneously, players’ accelerations and decelerations above 2.5 m•s^-2^ were recorded. This threshold has been previously used for medium and high intensity accelerations (Bradley et al., 2010; Dalen et al., 2021). Player Load was also measured. This variable is based on the acceleration data that are recorded by triaxial accelerometers integrated in the GPS devices and have been used to assess the neuromuscular load (Gomez-Carmona et al., 2019; Reche-Soto et al., 2019). Each player wore a heart rate monitor (Polar Team 2®, Polar Electro Oy, Finland) to obtain values for the maximal heart rate (HR_max_) reached during the SSG and the internal training load using the modified training impulse (TRIMP_mod_) proposed by Stagno et al. (2007). In order to determine the individual HR_max_ of each player, all the players completed a YYIRT1 before the study (Bangsbo et al., 2008). The rate of perceived exertion (RPE) was also measured (Foster et al., 2001). All players were fully familiarized with the use of the RPE before the beginning of the study.

### Design and Procedures

Data were collected from six training sessions during the 2020–2021 season (October–November). Measurements took place on the hardest day of the microcycle, when a higher neuromuscular load on the field was planned (Wednesday). One SSG was performed each training session. These sessions were performed on an artificial turf at the same time each day (16:00–18:00 pm) and begun with a 20-min standardized warm-up of mobility, active stretching and the same format and duration of passing drills.

**Figure 1:**
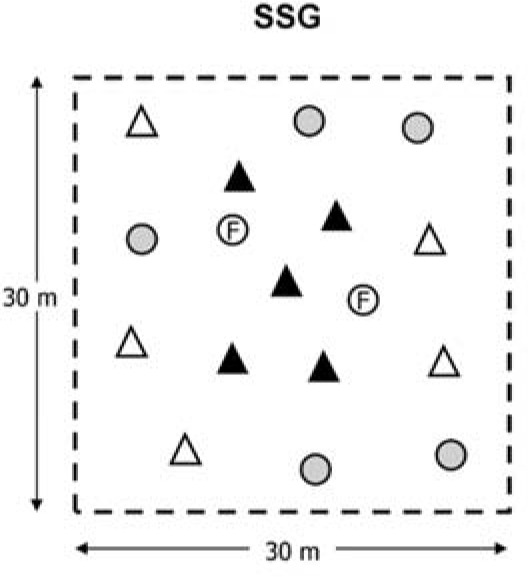
Graphical representation of the SSG studied.

The SSG format used in the study was 5 vs. 5 + 5 with 2 floaters (Figure 1), where two teams had to maintain ball possession, and one team had to recover it. When the defensive team intercepted the ball, or if during possession the ball left the field of play, a new ball was introduced by the technical staff, and the defensive team continued in the defensive role for the remaining duration of the task. The aim of the defensive team was to recover the highest number of balls during their defensive participation, while the offensive teams had to maintain ball possession. Depending on the defined period of 30 s (SSG_30_), 1 min (SSG_1_) or 2 min (SSG_2_), teams took a defensive role for these periods of time and changed roles when the defined period was completed. The SSG was played using a continuous bout of 12 min on a 30 x 30 m pitch. Each team completed one period of the defensive role and two periods of the offensive role continuously until finishing the bout. The area per player was of 75 m^2^ to ensure defensive technical-tactical aspects (Clemente and Sarmento, 2020; Fradua et al., 2013). SSG_30_ was performed on weeks 1 and 4, SSG_1_ on weeks 2 and 5, and SSG_2_ on weeks 3 and 6.

### Statistical Analysis

Data are presented as mean ± standard deviation (SD). The Shapiro-Wilk test demonstrated that all variables presented a normal distribution. Differences between players were determined using one-way analysis of variance (ANOVA). Post-hoc tests were calculated using Bonferroni correction for multiple comparisons to determine significant differences. The level of statistical significance was set at *p* ≤ 0.05. Standardized differences in effect size (ES, 95% confidence interval [95%CI]) in selected variables were calculated. The threshold values for assessing magnitudes of the ES (changes as a fraction or multiple of baseline standard deviation) were < 0.20, 0.20, 0.60, 1.2 and 2.0 for trivial, small, moderate, large and very large ES, respectively (Hopkins et al., 2009). SPSS (version 19, SPSS Inc., Chicago, IL, USA) was used for all statistical analyses.

## Results

**Figure 2:**
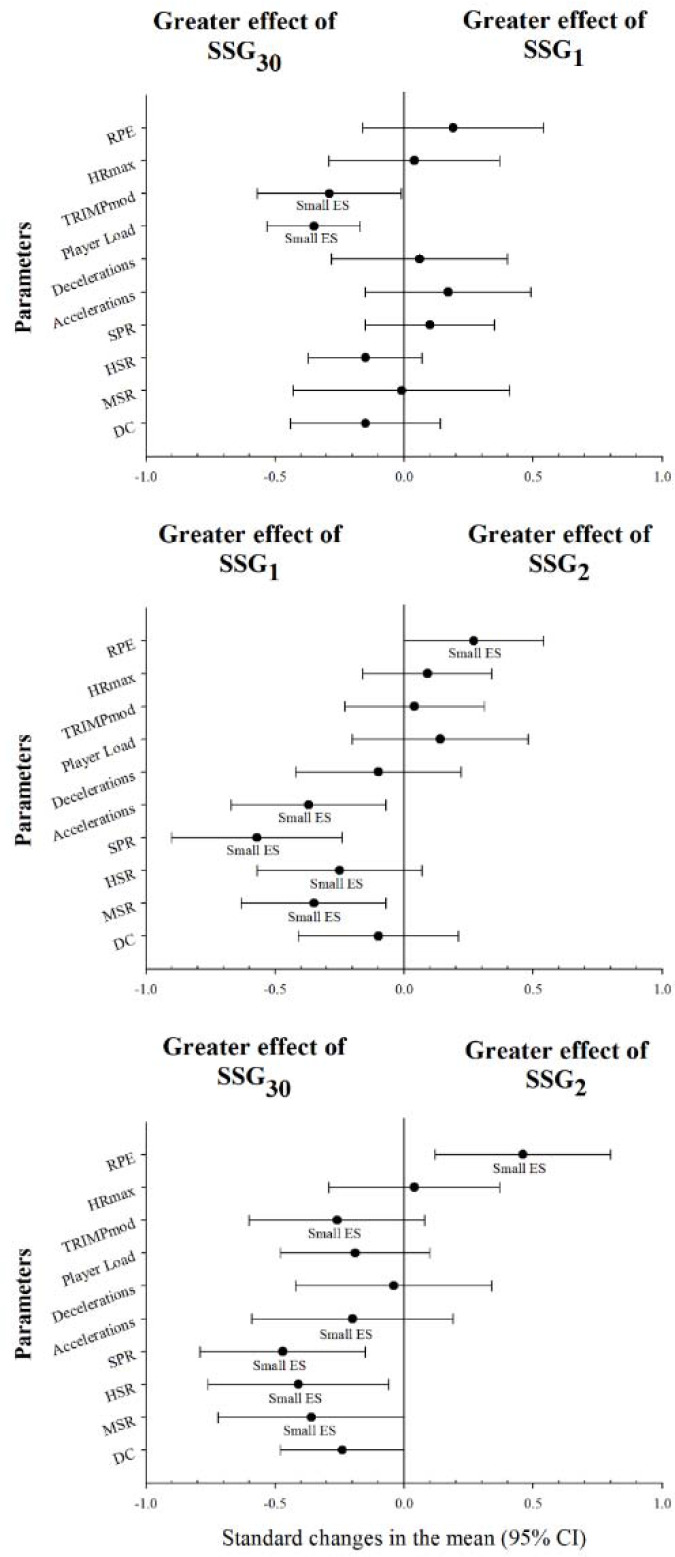
Comparison of external and internal loads in the SSG studied. DC = total distance covered; MSR = moderate speed running distance; HSR = high speed running distance; SPR = sprinting distance; TRIMPmod = modified training impulse; HR = heart rate; max = maximal; RPE = rate of perceived exertion; ES = effect size.

**Figure 3:**
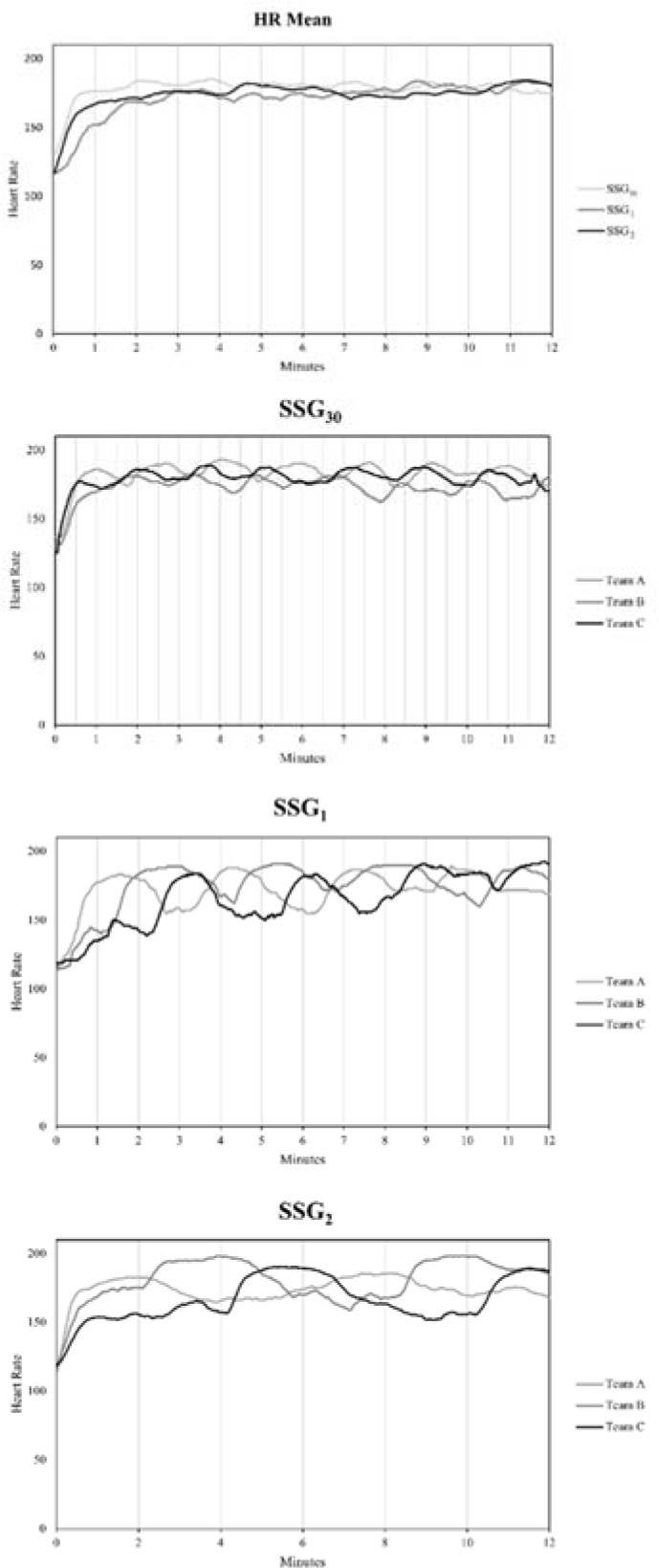
Comparison of the HR in the three fixed time periods of the defensive phase in the SSG format. *SSG = small-sided game; HR = heart rate*.

Table 1 shows the internal and external loads for the studied SSGs. Data analysis is presented in Figure 2, and revealed a small increase between SSG_30_ and SSG_1_ in Player Load (ES = −0.35 [−0.53 – −0.17]; *p* < 0.01). A small increase between SSG_30_ and SSG_2_ was observed in high speed running (ES = −0.41 [−0.76 – −0.06]; *p* < 0.05) and sprinting (ES = −0.47 [−0.79 – −0.15]; *p* < 0.01). Between SSG_1_ and SSG_2_, a small increase was shown in sprinting (ES = −0.57 [−0.90 – −0.24]; *p* < 0.01) and accelerations (ES = −0.37 [−0.67 – −0.07]; *p* < 0.05). In addition, SSG_2_ displayed a small increase in the RPE compared to SSG_30_ (ES = 0.46 [0.12–0.80]; *p* < 0.05). Comparison of the rest of variables yielded unclear differences. Figure 3 shows the HR responses in the drills played.

**Table 1:** Comparison of external and internal loads in the SSG studied.

	Mean ± SD			p value		
	SSG_30_	SSG_1_	SSG_2_	SSG_30_ vs. SSG_1_	SSG_30_ vs. SSG_2_	SSG_1_ vs. SSG_2_
DC (m)	1345.0 ± 115.4	1314.0 ± 148.9	1292.8 ± 159.4	0.402	0.099	0.596
MSR (m)	219.9 ± 80.8	207.8 ± 69.6	167.8 ± 54.9	0.982	0.100	0.038
HSR (m)	77.4 ± 44.2	52.3 ± 25.5	40.8 ± 24.5	0.242	0.049	0.187
SPR (m)	20.8 ± 22.1	17.5 ± 13.7	9.7 ± 12.7	0.514	0.021	0.001
Accelerations (#)	10.2 ± 3.5	11.0 ± 3.4	9.0 ± 3.1	0.375	0.387	0.043
Decelerations (#)	6.0 ± 2.7	6.0 ± 2.1	5.9 ± 2.8	0.773	0.845	0.592
Player Load (au)	159.3 ± 17.7	146.5 ± 14.2	153.9 ± 28.9	0.003	0.272	0.500
TRIMP_mod_ (au)	31,8 ± 7.3	28.4 ± 7.5	29.0 ± 7.9	0.091	0.212	0.817
HR_max_ (bpm)	191.4 ± 6.7	192.3 ± 8.7	193.4 ± 7.3	0.819	0.490	0.530
RPE (au)	7.6 ± 0.8	7.8 ± 0.5	8.0 ± 0.7	0.371	0.031	0.101

SD = standard deviation; DC = total distance covered; MSR = moderate speed running distance; HSR = high speed running distance; SPR = sprinting distance; HR = heart rate; max = maximal; RPE = rate of perceived exertion; au = arbitrary units; bpm = beats per minute.

## Discussion

The aim of this study was to evaluate the effects of different fixed time periods during the defensive phase on the internal and external loads of soccer players, during the same SSG. The results are partially in line with the hypotheses. Even though players’ responses during both SSG_30_ and SSG_1_ were very similar, players showed a small increase in the RPE and a small decrease in high speed running distance and sprinting distance in SSG_2_.

It has been determined that the duration of SSGs can influence the external load of soccer players (Halouani et al., 2014; Sarmento et al., 2018). Previous studies have shown that shorter duration induces greater total distance covered and more distance covered at high speed in SSGs (Casamichana et al., 2013; Clemente et al., 2019a; Hill-Haas et al., 2009). For example, Hill-Haas et al. (2009) compared a 1 x 24 min format with a 4 x 6 min format for three different SSGs, finding that players covered a significantly greater distance at high speed running in formats with shorter bouts. The present study found a small increase in high speed running distance during SSG_30_ and SSG_1_ in comparison with SSG_2_. However, total distance did not change in the different formats analysed. Several processes could explain these differences in players’ time-motion responses. The level of neuromuscular fatigue experienced by players during sets of 2 min may have been greater than during 1 min and 30 s bouts. It has been shown that fatigue during intense periods of match play is possibly related to intramuscular acidosis or the accumulation of potassium in the muscle interstitium (Mohr et al., 2005). It is possible that during SSG_2_ players experienced similar processes to those experienced in some intense periods of match play. Also, the longer periods proposed for SSG_30_ and SSG_1_ in which players continue playing in an offensive role (1 min and 2 min, respectively), but with more attackers (12 attackers vs. 5 defenders) could induce an active recovery and may be sufficient to facilitate recovery for involvement in high speed running actions. During matches, soccer players generally have a mean recovery time between high speed running bouts of 65–75 s (Bradley et al., 2009). This period of time has been shown to be sufficient for PCr re-synthesis and muscle re-oxygenation in repeated sprint studies (Brocherie et al., 2015; Padulo et al., 2015). On the other hand, a longer time in the defensive phase could induce a pacing strategy in players, resulting in less external loads due to awareness of the length of the defensive period and consequent self-organization of the pace (Sampson et al., 2015). By contrast, in shorter defensive play phases, the change of the role from offensive to defensive may induce aggressive behaviour related to ball recovery, and thus greater external loads. This behaviour may be strengthened by the numerical superiority of the offensive team, meaning that the defensive team may be working as a unit and waiting for the right moment to apply pressure (Nunes et al., 2020).

With regard to internal load responses, previous investigations have shown contradictory results. While some studies showed that longer formats produced higher heart rate and RPE values in comparison with shorter duration formats (Clemente et al., 2019a; Hill-Haas et al., 2009; Tessitore et al., 2006), another found that heart rate responses appeared to remain higher in a 4 x 4 min SSG format than in a 2 x 8 or a 1 x 16 min game, using a 5 vs. 5 SSG format (Casamichana et al., 2013). Our results showed no differences in TRIMP_mod_ and HR_max_ between SSGs when the defensive phase was 30 s, 1 min or 2 min. Probably, the minority transition between phases of play with specific periods of defence induced specific pacing strategies in the teams (Nunes et al., 2020), provoking a decrease in exercise intensity during this phase. Although our results did not show statistically significant differences in TRIMP_mod_ and HR_max_ between the SSG formats carried out, a small increase in the RPE was recorded by players during SSG_2_ in comparison with SSG_30_. These data are in line with the findings of previous studies, showing greater RPE values in longer duration formats of SSGs in comparison to shorter duration formats (Clemente et al., 2019b; Hill-Haas et al., 2009). Although the external load was greater in SSG_30_, and there were no differences in the internal training load in the three specified defence periods, neuromuscular fatigue effects may have been the cause of the increase in perceived exertion. This may apply specifically to SSG_2_, where players had to maintain their efforts to recover the ball for 2 minutes, producing a multidimensional fatigue effect because they had to perform defensive decision-making actions continuously (Smits et al., 2014).

To our knowledge, this is the first study to evaluate the effects of defensive SSGs in soccer players. Although this study provides new insights for designing specific training, some limitations must be acknowledged. First, although the differences found are statistically significant, the values shown by the ES are small. This could be due to the size of the sample, or the high variability of external and internal loads during SSGs (Clemente et al., 2022). Therefore, future studies should take these aspects into account. Only a format with offensive superiority (5 vs. 5 + 5 players with 2 floaters) was investigated, thus these data cannot be extrapolated to other formats. Previous research indicates that high-inferiority numeric situations during SSGs may induce minor running demands. This may be due to the fact that in situations with a high level of difficulty related to recovering the ball, players in the defensive phase perform conservative behaviours (Torres-Ronda et al., 2015). Future studies could analyse the consequences of defensive SSGs with numerical equality or lower numerical imbalances, and their suitability to reproduce the demands of soccer matches. Despite the fact that external and internal loads can be affected by the phase of play (Castellano et al., 2022), this study did not differentiate between the offensive and defensive phases. Future studies could focus on this issue to understand how the tactical context affects the training load. In addition, in this study there was no comparison made between defensive and traditional SSGs (where change of the phase is accomplished without using established periods), thus studies that investigate this comparison are needed.

The present investigation provides useful information for coaches about the duration of SSGs that constitute one of the main constraints in the daily design of training. Our results indicate that shorter fixed periods in defensive SSGs increased high speed running, while longer fixed periods resulted in greater perceived exertion. Manipulation of the time duration of defensive SSGs is another variable that should be considered during soccer training when these tasks are used. These findings suggest that practitioners could use shorter fixed periods in defensive SSGs with the aim to increase the external load of players in the strongest session of the microcycle. Furthermore, longer periods in defensive SSGs could be used to increase the fatigue effect of players. Therefore, coaches should avoid longer defensive periods in the days preceding the match.
